# Dual Career of the U-23 Spanish Canoeing Team

**DOI:** 10.3389/fpsyg.2019.01783

**Published:** 2019-08-02

**Authors:** Juan Gavala-González, Alfonso Castillo-Rodríguez, José Carlos Fernández-García

**Affiliations:** ^1^Department of Physical Education and Sports, University of Seville, Seville, Spain; ^2^Department of Physical Education and Sports, University of Granada, Granada, Spain; ^3^Department of Didactics of Languages, Arts and Sport, University of Malaga, Andalucía-Tech, Málaga, Spain; ^4^IBIMA, Málaga, Spain

**Keywords:** dual career, psychological well-being, canoeing, high-education, sport

## Abstract

Canoeing is one of the sport disciplines that brings great success to Spain in international competitions and Olympic Games (of the 17 medals won in Rio in 2016, four were in this sport, including three gold). However, the journey to become an elite athlete coincides in time with the challenge of pursuing an academic education, which often involves making difficult choices in the training-university dichotomy. The aim of this research was to ascertain how the Spanish under-23 calm water canoeing team perceives their athletic and academic careers. The present study was carried out with the Spanish under-23 flat water canoeing team, the step prior to competition at the highest level. The study sample comprised the whole population, namely the entire national team, made up of 21 athletes (11 women and 10 men) with a mean age of 20.57 ± 2.64 years and 10.00 ± 3.49 years of experience. These athletes are usually based at La Cartuja High Performance Center (Seville) and combine their sports activity with studying toward a university degree. A double qualitative and quantitative methodology was used. For the first of these, an interview script was elaborated based on the theoretical model by Wylleman et al. (2013). Applicable consents were requested from the Spanish Royal Canoeing Federation, coaches and paddlers. Interviews were carried out with each athlete twice: at the beginning and at the end of the season/academic year, which were recorded and subsequently tabulated and analyzed. Concerning quantitative methodology, the ESTPORT dual career questionnaire validated by Sánchez-Pato et al. (2016) was used. The results show that for all the paddlers canoeing is very important. It is typically complicated for them to attend classes, and subject planning is usually based on their training schedules. In the second/last part of the course, it is stressful for these athletes to combine both activities and some drop out of school. During the course, though, they appreciate having another activity that allows them to escape from the training routine. In addition, they miss not having an academic tutor to guide and advise them. These student-athletes are aware that their sport causes them to miss out on many moments with family and friends due to training or competing. However, at present this is offset, largely because of their high level of performance, which makes it easier for them to obtain scholarships that provide economic support.

## Introduction

The transition of any young person when he or she enters university is not an easy process, but when this must also be combined with training and competitions, this step becomes even more important (Tekavc et al., 2015). The dual career (DC) path undertaken by athletes involves the challenge of combining a career in sports with studies or work, a concern for most high-performance athletes (Ryba et al., 2015). In the case of student-athletes the optimal DC balance has been defined by Stambulova et al. (2015) as the combination of sport and studies that helps athletes achieve their academic and athletic goals and live a satisfying private life, maintaining their health and well-being.

It is well known that the practice of sport has been included in the strategic agenda of the European Union to promote integration among nations and cultures (European Commission, 2007). Nonetheless, when high levels of specialization and competition are achieved, the young people involved face the challenge of combining their athletic and educational preparation (Conzelmann and Nagel, 2003; Capranica and Millard-Stafford, 2011; Lobillo and Guevara, 2018), which quite often leads to their abandoning the practice of high-level sports in order to give priority to education with the aim of benefiting from future employment opportunities (Amara et al., 2004) or, conversely, to neglect academic preparation (Capranica and Millard-Stafford, 2011; Aquilina, 2013; Sohn and Hong, 2014; Guidotti et al., 2015; López de Subijana et al., 2015; Huang et al., 2016; Miro et al., 2017; Sum et al., 2017; Barriopedro et al., 2018; Krueger et al., 2018), resulting in low academic performance and limiting future and adequate inclusion in the working world (Conzelmann and Nagel, 2003; Kuettel et al., 2017; Vilanova and Puig, 2017; Barriopedro et al., 2018; López de Subijana et al., 2018).

All types of athletes are affected by the DC, regardless of social status, type of sport, location, the sex of the athlete or his or her experience (Erpič et al., 2004). According to Ryba et al. (2017), one of the main challenges faced by elite athletes between the ages of 12 and 25 is to successfully combine education and high-performance sport.

Early research (Purdy et al., 1982) indicated the negative effects of a double career on academic success. However, the most recent research suggests that talented athletes tend to perform well not only in sports, but also in the academic environment because they are highly motivated to perform well in both domains (Guidotti et al., 2015; Lupo et al., 2015; Fuchs et al., 2016; De Brandt et al., 2017; Isidori et al., 2017; Jordana et al., 2017; Kristiansen, 2017; Kuettel et al., 2017; Miro et al., 2017; Budevici-Puiu, 2018; Guirola et al., 2018; Kerstajn et al., 2018; Knight et al., 2018; Krueger et al., 2018; López de Subijana and Equiza, 2018; Ronkainen et al., 2018; Stefano and Ginevra, 2018; Tekavc and Erpic, 2018). Supporting this idea, O’Neill et al. (2013) concluded that student-athletes were determined to combine their education and sport, explaining that both dimensions strengthen their sense of identity, purpose and well-being.

Among athletes who abandoned their athletic careers due to different personal situations, a better transition and incorporation into working life was observed in those who had combined their double careers compared to those athletes who focused exclusively on their athletic careers (Aquilina, 2013; Torregrosa et al., 2015; Tshube and Feltz, 2015). Therefore, according to Stambulova and Wylleman (2015), to become long-term winners, student-athletes should first “win in the short term,” i.e., adapt well to their current DC programs for possible future benefits.

The DC guarantees that the athlete will have a future outside of sports when his or her high-level career ends (Stambulova and Wylleman, 2015), although it is true that during the years in which the athlete tries to combine sport and studies, this situation causes episodes of tension and anxiety (Gustafsson et al., 2008; Aquilina, 2013; O’Neill et al., 2013; Torregrosa et al., 2015; Sorkkila et al., 2017). For this reason, in some cases, the help and support of psychologists is needed to guide the athlete to successfully continue the DC (Stambulova et al., 2009; Mateos et al., 2010). Consequently, many athletes are convinced of the importance of combining the highest level of athletic life with academic life, especially in those minority sports that do not have sufficient financial support and/or their athletic life is short (Conzelmann and Nagel, 2003; Debois et al., 2015; Gledhill and Harwood, 2015; Kuettel et al., 2017; Barriopedro et al., 2018; López de Subijana et al., 2018; Pink et al., 2018). Aware of the consequences for athletes of combining two careers, many universities and organizations at the national and state levels have created support programs (Park et al., 2013; Brown et al., 2015; López de Subijana et al., 2015; Pink et al., 2015). Also relevant are the results of Barriopedro et al. (2018), which support the holistic vision of the development of the athletic career, showing an interrelation between the academic-vocational and the athletic areas, indicating the possible role of gender in the pursuit of both careers.

We see the opposite side of this situation with some athletes who are still not aware of the reality in which they live. Everything that is not related to their athletic career is indifferent to them or they do not give it the importance they should, which causes them to miss out on opportunities that exist outside their sport (Gorley et al., 2001; Lavallee and Robinson, 2007; Stambulova et al., 2009; Kelly and Hickey, 2010; Aquilina, 2013).

All athletes have their moment and it is at that point when a conscious change is noted that their athletic career is about to end and they search for a strategy to plan for the future (Park et al., 2012). According to the study by North and Lavallee (2004), it was observed that 79% of the athletes who sensed that in a few years their athletic career would come to an end, began to plan their future for that time, showing less interest in dedicating time to training and even defending the importance of work outside the realm of sport.

However, no studies currently exist on canoeing, one of the most internationally successful sports in Spain. We therefore consider this research to be innovative as the aim of our study was to gain insight into how the Spanish under-23 flat water canoeing team members (those who are called to take over from the paddlers in the international competitions) experience the duality between their athletic and academic careers.

## Methodology

### Participants

The study participants comprised 21 paddlers (10 men and 11 women) with a mean age of 20.57 ± 2.64 years y 10.00 ± 3.49 years of experience from the permanent pool of the Spanish Under-23 Flat Water Canoeing Team at CEAR La Cartuja (Specialized Center for High Performance Rowing and Canoeing) in Seville. All the paddlers, in addition to competing at an international level, are enrolled in a university degree program. Table 1 shows the characteristics of the participants. To avoid potential identification, areas of study were classified as either Sciences or Social Sciences.

**TABLE 1:** Characteristics of the participants.

**Athlete**	**Sex**	**Age**	**Years of experience**	**Specialty**	**University Degree**
1	Female	18	10	Kayak	Sciences
2	Female	24	12	Kayak	Social Sciences
3	Female	24	13	Kayak	Social Sciences
4	Female	19	7	Kayak	Social Sciences
5	Female	20	8	Kayak	Sciences
6	Female	18	9	Kayak	Sciences
7	Female	18	6	Kayak	Sciences
8	Female	23	7	Kayak	Social Sciences
9	Female	22	10	Kayak	Social Sciences
10	Female	27	19	Canoe	Social Sciences
11	Female	17	5	Canoe	Social Sciences
12	Male	23	14	Kayak	Social Sciences
13	Male	23	14	Kayak	Social Sciences
14	Male	19	9	Kayak	Sciences
15	Male	22	15	Kayak	Sciences
16	Male	19	5	Kayak	Social Sciences
17	Male	20	10	Kayak	Social Sciences
18	Male	19	9	Kayak	Social Sciences
19	Male	20	9	Canoe	Social Sciences
20	Male	19	9	Canoe	Social Sciences
21	Female	18	10	Kayak	Sciences

### Procedure

The methodology used was twofold: qualitative-quantitative. Consents were requested from the Royal Spanish Canoeing Federation, coaches and paddlers. Subsequently, individual contact was made with each athlete to arrange the date and time. In all cases, the place chosen was the facilities of CEAR La Cartuja, a space where all the athletes feel comfortable. The purpose of the study was explained to them and they were asked to consent to the use of the information, notifying them of the confidential nature and providing a brief explanation of the study. The interviews lasting between 10 and 15 min were recorded then later transcribed and analyzed-codified according to the levels of the theoretical model described. The five levels were analyzed according to two criteria, facilitators and barriers.

### Instruments

The qualitative portion used a semi-structured interview based on the theoretical model by Wylleman et al. (2013), which is based on three main themes: (a) balancing studies and athletic career; (b) balancing economic level and athletic career and complementary studies/activity; (c) balancing psychosocial level and athletic career and complementary studies/activity. This model is a reformulation of the original holistic model by Wylleman and Lavallee (2004) which defined four stages in the athletic career: initiation, development, mastery and discontinuation. According to the holistic model of the athletic career, not only should the athletic dimension be taken into account, but also other facets involved in the development of the person such as the individual, psychosocial, academic/vocational and financial aspects. In this way, the athlete can be better understood from an overall perspective. Despite sharing elements with previous models, the differential contribution of these authors was that of presenting the athletic stages in a coordinated manner together with psychological, psychosocial and academic-vocational stages thus contributing a holistic perspective to the concept of the athlete and defining both stages and transitions for each area, adding the economic dimension in the 2013 version. In our case, the athletes studied were classified according to the above-mentioned model at the level of mastery, as they are members of the permanent Spanish Canoeing Federation team; at the psychological level at the end of adolescence/beginning of adulthood; at the psychosocial level according to their relationship with peers, family, coaches and teammates, university classmates/professors; at the academic and vocational levels as a semi-professional or professional athlete and university student and finally at the economic level with financial support from government scholarships, sponsors, and family.

The interview was therefore structured around the three basic pillars established by Wylleman et al. (2013): balancing academic career with athletic career (Athletic Level and Academic/Vocational Level blocks); balancing psychosocial level with athletic and academic careers (Psychosocial Level block); and balancing economic level with athletic and academic careers (Economic Level block). The psychological level was left out since the purpose of this research was not to assess the level of psychological maturity of the subjects.

The quantitative methodology used was the DC questionnaire called ESTPORT validated by (Sánchez-Pato et al., 2016). This questionnaire is the result of a project approved by the European Commission in 2014 entitled the “Development of an Innovative European Sport Tutorship Model for the Dual Career of Athletes” ESTPORT the aim of which was “to develop, transfer and implement an innovative EU Sport Tutorship program in different European Universities” in order to comply with the EU’s DC guidelines for athletes. Therefore, due to these characteristics, this questionnaire was considered an appropriate tool and with great potential to be extrapolated to other countries.

This questionnaire was administered after the interview and completed in the presence of the interviewer. ESTPORT consists of two large blocks: the first on sociodemographic variables and the second on the DC, which in turn is divided into academic career and athletic career. The paddlers answered the questions in these blocks, which were structured into two types of answers: yes or no; or on a Likert scale ranging from very easily/totally agree, to very difficult/totally disagree, depending on the question.

### Data Analysis

The statistical software packages used were *SPSS* for *Windows v.23* (*IBM SPSS Statistics*, Chicago, IL, United States) and *Microsoft Office Excel (Microsoft Corp*., Redmond, WA, United States). To verify the normality of the variables, the *Kolmogorov–Smirnov* test was performed, and a reliability analysis was carried out using *Cronbach’s alpha* statistic of the closed-ended questions on the Likert scale, regarding the perceptual dimensions of academic career, athletic career and digital competence of the athlete. Subsequently, a *t*-test was conducted with the independent variable of sex and category (semi-professional or professional). The effect size (*d*) was also calculated, which quantifies the size of the difference between the two groups (Coe and Merino, 2003). Threshold values for the effect sizes on the ANOVA test were small, 0.10; moderate, 0.25; and large, 0.40 (Cohen, 1988). The level of significance was set at *p* < 0.05.

## Results

This study includes both quantitative and qualitative results.

### Quantitative Results

Table 2 shows the results of the reliability testing for the dimensions of academic career, athletic career and digital competence of the athlete. The dimensions evaluated show medium–high reliability values. Reliability data reported for the athletic career are scarce mainly due to the diversity in the type of response. In some cases, the responses were categorized as dichotomous values yes (1) and no (0), and in others, the answers were Likert type ranging from 1 to 5. This variety causes fluctuation in the reliability of the scale values, which suggests it would be preferable to use only one type of response. Moreover, in the academic career according to sex, reliability was lower because the perception of male athletes differs due to the type of university studies and the future professional career that they believe possible through these studies. In addition, an example of this was found in the categorization between semi-professionals and professionals, as it was observed that in the latter there were differing perceptions regarding their academic career, possibly due to the success achieved in their athletic career.

**TABLE 2:** Reliability of the psychological variables studied.

	***Cronbach’s α***				
	**All**	**Men**	**Women**	**Semi-professionals**	**Professionals**
Academic Career	0.590	0.478	0.836	0.944	0.624
Athletic Career	0.531	0.554	0.492	0.637	0.352
Digital Competence	0.871	0.711	0.714	0.723	0.661

To gain insight into how student-athletes perceive their DC, it is essential to verify the variance that exists between the populations studied. Table 3 shows the differences in perception between young men and women athletes of their academic career. There was little gender variance, with just two differences noted. The first was the distance between residence and university, with the female athletes living closer to the university than the male athletes. The second item corresponded to the importance of a better result, which the male athletes attached greater importance to than the female athletes. This lack of differences was due to the homogeneity of the sample, as they all practiced the same sport and were in a position to combine their athletic career with their university career.

**TABLE 3:** Descriptive statistics on the perception of university student high-level athletes of their academic career.

	**Item**	**Men (*n* = 10)**	**Women (*n* = 11)**	***T***	***P***	***d***
Flexible Curriculum	Item 21	2.90 ± 0.994	3.00 ± 0.894	−0.243	0.811	0.11
Distance learning	Item 22	2.56 ± 1.014	2.91 ± 0.944	−0.806	0.431	0.36
Univ. far from home	Item 26	4.10 ± 0.994	2.55 ± 1.440	2.849	0.010	1.25
Univ. far from training	Item 27	3.90 ± 0.994	2.91 ± 1.375	1.875	0.076	0.83
Studies ^∗^ training	Item 28	3.40 ± 1.506	3.27 ± 1.272	0.210	0.836	0.09
Employment ^∗^ studies	Item 29	2.44 ± 1.509	2.82 ± 0.982	−0.668	0.512	0.30
Caring for family	Item 30	2.13 ± 1.246	2.09 ± 1.446	0.054	0.958	0.03
Habitual tiredness	Item 31	3.90 ± 0.994	3.82 ± 1.328	0.158	0.876	0.07
I lose course rhythm	Item 32	3.70 ± 0.949	3.82 ± 0.874	−0.297	0.770	0.13
I lose contact with peers	Item 33	3.20 ± 1.476	2.91 ± 0.944	0.544	0.593	0.23
High cost of studies	Item 34	3.78 ± 1.202	3.00 ± 1.342	1.350	0.194	0.61
University support	Item 35	4.10 ± 0.994	3.73 ± 1.348	0.715	0.484	0.31
Inflexible study schedule	Item 36	4.50 ± 0.707	3.91 ± 1.375	1.218	0.238	0.54
Inflexible training schedule	Item 37	2.50 ± 1.434	3.09 ± 1.514	−0.916	0.371	0.40
Virtual tools	Item 44	2.90 ± 1.449	3.18 ± 1.168	−0.493	0.628	0.21
Importance of studies	Item 45	4.40 ± 0.966	4.55 ± 1.214	−0.302	0.766	0.14
Time to improve grades	Item 46	3.70 ± 1.160	3.55 ± 0.820	0.355	0.726	0.15
Usefulness of studies	Item 47	4.30 ± 0.949	4.55 ± 0.934	−0.597	0.558	0.27
Satisfaction with studies	Item 48	2.30 ± 1.418	2.27 ± 0.786	0.055	0.957	0.03
Capability for high grades	Item 49	1.90 ± 1.287	2.82 ± 1.401	−1.559	0.136	0.68
Earn university degree	Item 50	4.60 ± 0.843	4.73 ± 0.467	−0.433	0.670	0.19
Importance of best result	Item 51	4.70 ± 0.675	3.73 ± 1.104	2.405	0.027	1.06
Course content	Item 52	3.50 ± 1.434	4.09 ± 1.375	−0.964	0.347	0.42
Reason for university degree	Item 53	3.30 ± 1.636	3.09 ± 1.136	0.343	0.735	0.15
Effort for qualifications	Item 54	2.50 ± 1.434	2.09 ± 1.375	0.667	0.513	0.29
Difficult assignments	Item 55	3.00 ± 1.414	3.09 ± 1.514	−0.142	0.889	0.06
Studies knowledge and skills	Item 56	4.20 ± 1.135	4.55 ± 0.934	−0.764	0.454	0.34
Importance of univ. degree	Item 57	4.20 ± 1.135	4.55 ± 0.820	−0.805	0.431	0.35
University degree ^∗^ work	Item 58	4.10 ± 1.101	4.27 ± 0.905	−0.394	0.698	0.17

*Univ., university.*

The perception of the sports career of the athletes participating in the study was similar according to gender (Table 4). Regarding digital competence, the men used Twitter teaching support more frequently than the women (*P* < 0.05). In addition, the item of use of assignments as teaching support was marginally different (*P* = 0.06), with a higher frequency in the women.

**TABLE 4:** Descriptive statistics of the perception of university student high-level athletes of their athletic career and digital competence.

**Athletic career**	**Item**	**Men (*n* = 10)**	**Women (*n* = 11)**	***T***	***P***	***d***
DC sports monitoring	Item 59	2.40 ± 1.265	2.36 ± 0.924	0.076	0.940	0.04
Weekly training sessions	Item 60	9.80 ± 1.135	9.55 ± 0.820	0.593	0.560	0.25
Hours of weekly training	Item 61	4.70 ± 0.483	4.36 ± 0.924	1.028	0.317	0.45
Help with studies	Item 62	3.20 ± 1.476	2.73 ± 1.272	0.788	0.440	0.34
Training and academic performance	Item 63	4.10 ± 1.287	4.45 ± 0.688	−0.798	0.435	-0.34
Coordinate studies	Item 72	1.40 ± 0.894	1.57 ± 0.976	-0.310	0.763	−0.18
Final expectations	Item 73	1.67 ± 1.211	1.20 ± 0.447	0.811	0.438	0.53
**Digital competence**						
Virtual campus	Item 65	3.56 ± 1.333	4.27 ± 1.348	−1.189	0.250	0.53
Forums	Item 66	2.11 ± 0.601	2.45 ± 1.635	−0.596	0.559	0.28
Assignments	Item 67	2.78 ± 1.202	3.91 ± 1.300	−2.002	0.061	0.90
Chat	Item 68	2.63 ± 1.598	2.18 ± 1.401	0.642	0.529	0.30
Skype	Item 69	1.44 ± 0.527	1.73 ± 1.348	−0.591	0.562	0.28
Facebook	Item 70	2.11 ± 1.364	2.18 ± 1.537	−0.108	0.916	0.05
Twitter	Item 71	2.33 ± 1.323	1.27 ± 0.647	2.348	0.031	1.02

*DC, dual career.*

In the athlete’s perception of his or her academic career according to category (semi-professional or professional), there were once again two significant differences (Table 5). The first of these was regarding the time taken away from studying by their other tasks as an athlete, on which the semi-professional athletes scored 1.83/5 points, indicating that sport-related activities did not interfere with their academic work (*P* < 0.05; *d* = 1.08). The second item with the greatest variance was losing contact with their peers, on which the professional athletes had a better score (approaching disagreement), thus positively assessing their DC (*P* = 0.05; *d* = 1.02).

**TABLE 5:** Student’s *t*-test score by academic career category.

	**Item**	**Semi-professional (*n* = 6)**	**Professional (*n* = 15)**	***T***	***P***	***d***
Flexible Curriculum	Item 21	2.50 ± 1.049	3.13 ± 0.834	−1.464	0.159	0.70
Distance learning	Item 22	2.80 ± 0.447	2.73 ± 1.100	0.130	0.898	0.08
Univ. far from home	Item 26	3.67 ± 1.033	3.13 ± 1.598	0.904	0.381	0.40
Univ. far from training	Item 27	3.50 ± 1.378	3.33 ± 1.291	0.262	0.796	0.13
Studies ^∗^ training	Item 28	2.50 ± 1.643	3.67 ± 1.113	−1.896	0.073	0.83
Employment ^∗^ studies	Item 29	1.83 ± 0.983	3.00 ± 1.177	−2.123	0.048	1.08
Caring for family	Item 30	1.50 ± 0.837	2.38 ± 1.446	−1.382	0.185	0.74
Habitual tiredness	Item 31	3.17 ± 1.472	4.13 ± 0.915	−1.836	0.082	0.78
I lose course rhythm	Item 32	3.83 ± 0.408	3.73 ± 1.033	0.227	0.823	0.13
I lose contact with peers	Item 33	3.83 ± 0.983	2.73 ± 1.163	2.036	0.050	1.02
High price of studies	Item 34	3.00 ± 1.225	3.47 ± 1.356	−0.681	0.505	0.36
University support	Item 35	4.00 ± 0.894	3.87 ± 1.302	0.228	0.822	0.12
Inflexible study schedule	Item 36	4.33 ± 1.033	4.13 ± 1.187	0.360	0.722	0.18
Inflexible training schedule	Item 37	2.33 ± 1.033	3.00 ± 1.604	−0.936	0.361	0.50
Virtual tools	Item 44	3.00 ± 1.549	3.07 ± 1.223	−0.105	0.918	0.05
Importance of studies	Item 45	4.17 ± 1.169	4.60 ± 1.056	−0.826	0.419	0.39
Time to improve grades	Item 46	4.00 ± 1.095	3.47 ± 0.915	1.143	0.267	0.53
Usefulness of studies	Item 47	4.50 ± 0.548	4.40 ± 1.056	0.218	0.830	0.12
Satisfaction with studies	Item 48	2.33 ± 0.816	2.27 ± 1.223	0.122	0.904	0.06
Capability for high grades	Item 49	2.33 ± 1.506	2.40 ± 1.404	−0.096	0.924	0.05
Earn university degree	Item 50	4.83 ± 0.408	4.60 ± 0.737	0.725	0.477	0.39
Importance of best result	Item 51	4.50 ± 0.548	4.07 ± 1.163	0.865	0.398	0.47
Course content	Item 52	3.67 ± 1.751	3.87 ± 1.302	−0.289	0.776	0.13
Reason for university degree	Item 53	2.67 ± 1.506	3.40 ± 1.298	−1.120	0.277	0.52
Effort for qualifications	Item 54	2.17 ± 1.169	2.33 ± 1.496	−0.243	0.810	0.12
Difficult assignments	Item 55	3.00 ± 1.095	3.07 ± 1.580	−0.094	0.926	0.05
Studies knowledge and skills	Item 56	4.50 ± 0.837	4.33 ± 1.113	0.330	0.745	0.17
Importance of univ. degree	Item 57	4.50 ± 0.837	4.33 ± 1.047	0.347	0.733	0.18
University degree ^∗^ work	Item 58	4.33 ± 1.211	4.13 ± 0.915	0.413	0.684	0.19

*Univ., university.*

On the perception of athletic career and digital competency (Table 6) by category, no significant differences were observed, despite the fact that the effect sizes (Cohen’s *d*) were large in items 72 and 73 (*d* = 0.88 y 0.84, respectively) regarding athletic career and the use of chat to support teaching (*d* = 0.73).

**TABLE 6:** Student’s *t*-test score by category of athletic career and digital competency.

**Athletic career**	**Item**	**Semi-professional (*n* = 6)**	**Professional (*n* = 15)**	***T***	***P***	**d**
DC sports monitoring	Item 59	2.00 ± 1.265	2.53 ± 0.990	−1.032	0.315	0.50
Weekly training sessions	Item 60	9.67 ± 1.033	9.67 ± 0.976	0.000	1.000	0.00
Hours of weekly training	Item 61	4.33 ± 1.211	4.60 ± 0.507	−0.728	0.476	0.29
Help with studies	Item 62	3.17 ± 1.329	2.87 ± 1.407	0.448	0.659	0.22
Training and academic performance	Item 63	4.00 ± 1.549	4.40 ± 0.737	−0.815	0.425	0.33
Coordinate studies	Item 72	1.00 ± 0.000	1.60 ± 0.966	−1.964	0.081	0.88
Final expectations	Item 73	1.00 ± 0.000	1.63 ± 1.061	−1.667	0.140	0.84
**Digital competence**						
Virtual campus	Item 65	4.40 ± 1.342	3.80 ± 1.373	0.850	0.406	0.44
Forums	Item 66	2.20 ± 1.304	2.33 ± 1.291	−0.200	0.844	0.10
Assignments	Item 67	3.80 ± 1.789	3.27 ± 1.223	0.754	0.460	0.35
Chat	Item 68	3.25 ± 1.708	2.13 ± 1.356	1.393	0.181	0.73
Skype	Item 69	1.60 ± 0.894	1.60 ± 1.121	0.000	1.000	0.00
Facebook	Item 70	2.00 ± 1.732	2.20 ± 1.373	−0.265	0.794	0.13
Twitter	Item 71	2.00 ± 1.732	1.67 ± 0.900	0.567	0.578	0.24

*DC, dual career.*

### Qualitative Results

For a better understanding of the qualitative results, we established two blocks: circumstances that enable the athlete to maintain continuity between both careers: athletic and academic, which we call “facilitators,” and by contrast, other reasons that lead the athlete to choose just one of the careers that we call “barriers.”

#### Facilitators

Concerning the “facilitators,” in the athletic realm we note that that the coaches themselves are concerned about the need and importance of the academic education of their paddlers. In fact, they view the DC as positive because this way they also think about other things, in fact E1 commented: “*It is better that they study, that they interact with other people who have nothing to do with canoeing. This way they have other things to think about…if not, if one day training goes poorly, they will be thinking about how badly they did it…if they have to think about having to go to the Faculty, etc. they forget about it sooner,” “the things in the water stay in the water and have to be fixed in the water, driving themselves crazy about it only makes it worse.”*

Consistent with these words, the coaches have scheduled start times for each of the daily training sessions, although all paddlers say that coaches do not impose an obligation to attend each day’s training, and even tend to modify training schedules so that their paddlers can combine going to class and training.

P6 Comments: *“My coach, whenever possible, tries to help us., sometimes he lets us train later or earlier, but it’s not good to train every day alone either. Besides, he doesn’t force us to train, but if we push it, he reminds us that we are here thanks to a scholarship from the Federation.”*

On the psychological level, all canoeists comment on how important canoeing is and has been for them, and that, today, they do not understand and would not understand their lives without this sport. At the beginning it was the social environment that stood out (P11: *“I liked most the atmosphere, my teammates, I enjoyed training at the club, I had fun”*). Whereas, today, what motivates them is to be where they are and to be able to have it all. (P2: *“I love the sensation and the appetite for more. I’m very much a perfectionist and wanted to achieve more things”)*.

In relation to the psychosocial aspect, the affective part: surrounding themselves with their loved ones and their friends, this is their best time and although they have little time, and many see friends and relatives very occasionally, those days serve to “recharge batteries, escape and draw energy to continue training and their day to day” P14.

P19 comments: *“The basic pillar in all of this are friendships and your group of friends, if not, you go crazy. Family is fundamental. You need to clear your head on the weekends, get out a little bit, be with different people.”*

The academic level causes them the most distress, since attending class is very complicated as it usually coincides with training. So what they usually do is organize themselves at the beginning of the course/season. They ask their coach about the training schedules, and based on the schedule published in the faculty, they register for the courses they think they will be able to attend.

P3: *“In September, when I arrive here, I ask the coach about the timetables and I outline the practical classes I can attend, since the theoretical ones are not compulsory, if I can attend, I go, but the other ones, I enroll because I can attend class. I try to maintain this throughout the course. although when the end of the course arrives, which coincides with the peak of the (sports) season, I spend weeks without going to the University, I’m dead!”.*

In addition, peer support is essential to address this situation.

P8: *“*…*Having classmates who understand your situation and support you in everything (notes, group work, exams) is priceless!! To them, I owe half my degree.”*

P10: *“My classmates know that I can’t always go to class, they leave me their notes or let me know about important things the teacher has said. That way I can keep up to date. They also count on me for group work because they know that I need it so that I don’t fall behind in the subject.”*

Currently, the economic aspect is not an obstacle for these paddlers since most have a maintenance grant from the Federation. In addition, some meet the requirements for other grants.

P16: *“There are scholarships to help you pay the university tuition and if you can get them it is very helpful. I also usually apply for sports scholarships based on the results. In my case, the government of my region doesn’t give me anything, but there are other colleagues who receive other assistance because they are Basque or Galician*…*”.*

P7: *“I applied for a grant from the ministry, which I imagine they will give me. I have also just applied for the Galician high-level athlete grant and in theory they have to give me financial aid.”*

#### Barriers

##### Athletic

In general, for all the paddlers on the under-23 team who combine studies and training, both commitments overlap in time. Training schedules usually coincide with class schedules, so fulfilling both obligations is impossible. The most common option is to attend only the required classes (practical) and when these overlap, change the training schedule, even if this means training alone (without teammates or coach).

P13: “*Practically it is the day to day… mainly the practical classes usually coincide with training. I have to train here very early to arrive just in time for class or late and the truth is that it makes everything difficult for me because I arrive at class tired. it is hard to manage…at the beginning of the season, I can still…but when the peak season comes. I am just dead when I get out of the water and cannot even climb the ladder, so just imagine going to the University.*’

This academic-athletic situation affects the athlete at the psychological level with a considerable load of stress, since, as we have seen, being able to combine both activities on a daily basis is quite a complicated task.

P15: *“Since practice is in the morning and the afternoon, I try to squeeze everything into a single session (I know I lose training quality and don’t get enough rest between one and the other), but either I race against the clock or I can’t manage.”*

P17: *“I finish training between 1:00 and 2:00 p.m., I get home like at 3:30p.m. I take the bike to go to the university from 4:00 to 8:00 p.m., you get home and you have to start studying because if not, you have no other time to do it.”*

P11: *“I eat in a hurry because at 3:00 pm I have to leave to go to class, which I have from 4:00 to 8:00 p.m., when I come back to CEAR I stay there studying, doing homework or reading a little and at about 12:00 a.m. I go to sleep.”*

As we can see, in the life of the under-23 paddler there is no time for anything other than training, going to class, eating and resting. In this sense, the psychosocial aspect is the most neglected, certainly not because they want to, but because of lack of time. The most surprising thing is that they accept it and as it stands today their sacrifice is compensated.

P1: “*After so much time maintaining this rhythm it’s something I’ve internalized, but it’s complicated to handle, I sacrifice many moments with friends and family. Lose many weekends, holidays, birthdays, and even evenings with friends to disconnect. You have to devote many hours to training, rest if you want to achieve something in this…and in your free time, study.*”

P11: *“Friends are those who are in Galicia, I see them at Christmas or summer and that’s it.”*

P12: *“It’s very complicated, because at the Faculty they tell me to stay for a drink, but for me, I would love to stay, but I have to train, I have to complete a training session, and maybe I could after dinner, around 9.30 at night, but the next day at 7:00 a.m. it wouldn’t be realistic. so you have to be clear about what you are here for.”*

##### Academics

The academic realm presents the most barriers, and in greater number as the course and season progresses: attendance at classes, practical work, group work, exams…to which we should add training, gatherings outside Seville and the competitions, in many cases international. Successfully combining academic and athletic life becomes impossible even with good organization and, most of the time, one of the two facets is given up due to incompatibility. In all cases, it is academic life that is abandoned because the current university system does not benefit these athletes. In fact, many of them have chosen to switch from physically attending university to distance learning because of all these issues, while others have dropped out of university completely.

P3: *“I started at the Complutense University of Madrid, when the Senior Lady’s team was based at the Joaquín Blume residence in Madrid, and the following year when they told us that the training location would be changed, I decided to reconsider because the training location would be a bit volatile and I’m not going to be able to advance normally.”*

P19: *“The first year I only passed one subject and last year I repeated the first year and passed a few more, I saw that it was impossible and that I was not going to be able to manage it if I wanted to continue training at a high level and I was not going to be able to pursue a career in Medicine, it is a career that you have to dedicate all your time to and be very focused, I loved it, I thought it was amazing but with this sport and the level at which I do it, for me, at least it is impossible.” “The second quarter of the first year was very complicated. In the morning it was impossible to go to class. I trained every morning which is class time that you cannot change and that was the most complicated part really. Without going to class it is almost impossible to complete a career of that caliber.”*

Finally, from an economic standpoint, while there are scholarships to help these athletes, very few of them benefit from an athletic scholarship since they only receive one if they have been successful in international competitions and that is usually very difficult. These athletes spend years training at the highest level but are not assured that they will have compensation that will enable them to support themselves and to achieve independence. Some of them comment that they have had to look for an apartment outside the High Performance Center, because due to poor results in the previous year, they were not granted a scholarship, even though they are still on the team. Therefore, they need economic support from their family.

P13: *“I don’t receive any help or sponsorship, not from the club, or from the Asturian federation, and I don’t any opportunity for a scholarship to the University because I’m not registered for the whole course, so I’ m not entitled to it.”*

P5: *“Canoeing in itself is not a sport that makes it easy for you on an economic level: you often have to pay for a competition yourself or if the Federation does not pay for the physiotherapist, you have to pay for the treatment yourself. Then regarding studies there are more and more scholarships, but there are no scholarships linking athletes with their studies, many scholarships are awarded for getting very good grades or for passing everything, and of course an athlete does not have the same chance as a normal student to pass everything or to get very good grades.”*

These are other reasons why the paddlers admit that they cannot quit studying because they know that the day they do not get results, they will have to find another way to make a living, and without a university degree, the options are very scarce.

P9: *Who lives from canoeing in Spain? Maybe just one or two, and that’s because they are civil servants, and they are able to train because they have facilities at work. I’m doing this to get to Tokyo 2020, to have the experience of an Olympic Games, not because it compensates financially.”*

## Discussion

The purpose of the present study was to gain an understanding of the perception of life both in the athletic and the academic careers of the Spanish under-23 flat water canoeing team. The athletes showed some significant differences according to gender. The men live farther away from the university area and the sports facilities where they carry out their DC, an issue that may be related to their economic situation. Sometimes, when the problems are perceived by both populations, it is due to the university campus as well as the location where the training sessions are held frequently being located on the outskirts of the city. In addition, it is more difficult for the men to achieve a better result in their academic careers. This greater difficulty shown by the men may be associated with the constant concern that these athletes have for their future outside of sport (Stambulova and Wylleman, 2015), which translates into greater tension and anxiety (Gustafsson et al., 2008; Aquilina, 2013; O’Neill et al., 2013; Torregrosa et al., 2015; Sorkkila et al., 2017). Concluding the academic section according to gender, of note is that all the male athletes showed high scores on earning a university degree, the association between a university degree and finding work and having knowledge and skills, which indicates that they perceive excessive difficulty in achieving these goals. These concerns or difficulties demonstrate the need to incorporate tutors at universities for high-level athletes to help and support these athletes providing optimal academic guidance (Stambulova et al., 2009; Mateos et al., 2010). The perception of the sports career, however, showed no significant differences according to gender.

These athletes were classified according to their level of expertise, differentiating between professionals and semi-professionals. The former showed greater difficulty and confirmed that training time takes time away from their studies, leading to the conclusion that it is more challenging for them to combine their studies with their athletic career. In contrast, the semi-professionals claimed that training time causes a loss of contact with peers. Some studies corroborate that many athletes, especially in minority sports, are aware of the importance of combining athletic life at the highest level with academic life, mainly due to the fact that athletic careers are short (Conzelmann and Nagel, 2003; Debois et al., 2015; Gledhill and Harwood, 2015; Kuettel et al., 2017; Barriopedro et al., 2018; López de Subijana et al., 2018; Pink et al., 2018).

On a qualitative level, we have seen the shift in research on the DC since the early studies by Purdy et al. (1982) advised against it because of poor academic results, while more current studies (Guidotti et al., 2015; Lupo et al., 2015; Stambulova and Wylleman, 2015; Fuchs et al., 2016; De Brandt et al., 2017; Isidori et al., 2017; Jordana et al., 2017; Kristiansen, 2017; Kuettel et al., 2017; Miro et al., 2017; Budevici-Puiu, 2018; Guirola et al., 2018; Kerstajn et al., 2018; Knight et al., 2018; Krueger et al., 2018; Ronkainen et al., 2018; Stefano and Ginevra, 2018; Tekavc and Erpic, 2018) corroborate the desirability of a double life (academic and athletic), with which our results coincide.

Studies by Stambulova et al. (2015) suggest that the optimal balance in the DC is the combination of sport and studies, whereas Gustafsson et al. (2008), Aquilina (2013), O’Neill et al. (2013), Torregrosa et al. (2015), Ryba et al. (2017), and Sorkkila et al. (2017), warn of the complexity of successfully combining these two spheres, causing anxiety, stress and tension. This has also been verified in our study, and when these appear, the paddlers abandon their academic career, first partially (they only attend the required classes) and if their stress is very high, they drop out completely, which is consistent with the research by Purdy et al. (1982).

Concerning the economic aspect, our results agree with those of other studies such as the results by Conzelmann and Nagel (2003), Debois et al. (2015), Gledhill and Harwood (2015), Kuettel et al. (2017), Barriopedro et al. (2018), López de Subijana et al. (2018), and Pink et al. (2018) since our paddlers, despite having a maintenance grant, are aware that they must have an academic career, as no matter how successful a sporting career may be, they will never be able to earn a living from it.

This study has several limitations that must be taken into account. First, the questionnaire validated by a group of Spanish researchers in a sample of Spanish athletes appears to have discrepancies concerning the reliability and validity of the elements that comprise the sport category. Experts have proven that some of the items that refer to the knowledge of applications and use of social networks should not be considered in the athletic career category. Accordingly, we view this as a limitation and thus propose designing a new questionnaire that includes all the variables and categories in a comprehensive manner. Furthermore, it could be very interesting to consider differences in other types of sports, whether team, opponent or individual. Athletes in team sports, for example, are thought to have different perceptions of their DC, enabling variations to be found in items that can be improved or palliated due in part to a process of intervention, tutoring or mentoring, embedded within the university itself.

## Conclusion

This is the first study to describe the perception of the DC in both professional and semi-professional under-23 canoeists. This information makes it possible to verify the difficulties currently encountered by these paddlers. According to the data extracted from the results section of the qualitative methodology, the athletes encounter specific problems that make it difficult to efficiently combine their DC. Due to the existence of these complications, we recommend the incorporation of the position of a tutor for high-level athletes into the university system to support, help and guide athletes during the times they need greater attention.

## Data Availability

All datasets generated for this study are included in the manuscript and/or the supplementary files.

## Ethics Statement

This study was performed in compliance with the 1964 Declaration of Helsinki, revised in 2013, which defines the ethical guidelines for research involving human subjects. Prior to the study, written informed consent was obtained from the participants and their legal guardians. Throughout the entire research process and afterward, the provisions of the Organic Law 15/1999 of 13 December, on the protection of personal data were applied. All the participants were treated following the ethical guidelines of respect, confidentiality and anonymity in data processing.

## Author Contributions

All authors listed have made a substantial, direct and intellectual contribution to the work, and approved it for publication.

## Conflict of Interest Statement

The authors declare that the research was conducted in the absence of any commercial or financial relationships that could be construed as a potential conflict of interest.
